# Incorporation of Chitosan Nanoparticles into a Cold-Cure Orthodontic Acrylic Resin: Effects on Mechanical Properties

**DOI:** 10.3390/biomimetics6010007

**Published:** 2021-01-15

**Authors:** Mostafa Shahabi, Sorour Movahedi Fazel, Abdolrasoul Rangrazi

**Affiliations:** 1 Department of Orthodontics, School of Dentistry, Mashhad University of Medical Sciences, Mashhad 9177948959, Iran; shahabim@mums.ac.ir; 2 School of Dentistry, Mashhad University of Medical Sciences, Mashhad 9177948959, Iran; dr.srour.movahedi@gmail.com; 3 Dental Research Center, Mashhad University of Medical Sciences, Mashhad 9177948959, Iran

**Keywords:** mechanical properties, acrylic resin, chitosan, nanoparticles

## Abstract

Improvement of the antibacterial properties of acrylic resins, used in the construction of removable orthodontic appliances, is an important strategy to reduce the incidence of caries and oral diseases in orthodontic treatments. The addition of antimicrobial agents to acrylic resins is one of the effective methods to enhance the antimicrobial properties of these materials. However, one main concern is that modification of acrylic resin has negative effects on its mechanical properties. Recently, chitosan nanoparticles (NPs), as biocompatible and biodegradable polysaccharides with remarkable antimicrobial properties, have been used in different areas of dentistry and medicine. This study aimed to investigate the effects of adding chitosan NPs on the mechanical properties of a cold-cure orthodontic acrylic resin. The chitosan NPs were added to the acrylic resin in various weight percentages: 0% (control), 0.5%, 1%, 2%, and 4%. The flexural strength, compressive strength, Vickers microhardness, and impact strength measurements were performed for all five groups. The results showed that adding up to 1% (*w/w*) chitosan NPs to an acrylic resin had no significant negative effects on its flexural strength and compressive strength, while it decreased these parameters at weight percentages of 2% and 4% (w/w). The results also revealed that modification of acrylic resin with chitosan NPs up to 4% had no significant negative effects on the microhardness and impact strength of acrylic resin. In conclusion, the addition of chitosan NPs up to 1% (*w/w*) had no significant negative effects on the mechanical properties of cold-cure acrylic resin.

## 1. Introduction

The growing demand for orthodontic treatments has led to an increasing need for the use of orthodontic acrylic resins in the fabrication of removable orthodontic appliances and retainers. Accumulation of microorganisms on acrylic resins is one of the important challenges in the use of these materials. Poor oral hygiene in orthodontic patients and surface porosities are two factors that lead to the accumulation of residual foods and microorganisms, such as *Streptococcus mutans* and *Candida albicans* on acrylic resins. The accumulation of these microorganisms increases the incidence of caries and oral diseases and jeopardizes the efficiency of orthodontic treatments [1]. This is especially important for cold-cure acrylic resins that have a higher porosity than heat-cure acrylic resins [2].

Mechanical and chemical methods, as well as their combinations, are suggested for cleaning acrylic resins in removable orthodontic appliances. So far, various chemical disinfectant solutions have been used to eliminate oral microorganisms from acrylic resins. However, several studies have shown that these disinfectants exert negative effects on the mechanical and physical properties of acrylic resins, such as flexural strength [3], roughness [4,5], hardness [6,7,8], and color [3,9]. On the other hand, chemical and mechanical methods, such as manual brushing with a toothbrush, depend on patient cooperation.

In recent years, researchers have focused on preventive methods that do not require patient cooperation [10]. Addition of antimicrobial agents to dental materials is one of the effective strategies to enhance the antimicrobial properties of these materials. Although this method is independent of the patient’s collaboration, it is important to make sure that this modification has no significant negative effects on the mechanical or physical properties of the material. It is preferable to use nanosized antibacterial agents, because they have a greater surface-to-volume ratio, have intimate interactions with microbial membranes, and provide a considerably larger surface area for antimicrobial activity [11].

Several metal nanoparticles (NPs), such as silver, zinc oxide (ZnO), titanium dioxide (TiO_2_), and copper oxide (CuO) NPs, have been used for modification of orthodontic dental materials [12,13,14,15], although the use of biocompatible non-metal NPs is more preferable. In recent years, chitosan, as a natural, non-toxic, biocompatible, and biodegradable polysaccharide with remarkable antimicrobial properties [16,17], has been used in different areas of dentistry, such as modification of restorative dental materials, adhesion and dentin bonding, enamel repair, and modification of dentifrices [18]. Chitosan is a cationic material, as it contains one primary amine group. This polysaccharide can adhere to the bacterial cell wall and degrade its structure, as well as the cell membrane of bacteria [19,20]. Nanosized chitosan also exhibits superior antimicrobial activities [20].

Chitosan NPs can be added to acrylic resins to improve their antibacterial properties. Generally, it is important to make sure that incorporation of chitosan NPs into acrylic resins does not exert any significant adverse effects on their mechanical properties. To the best of our knowledge, the effect of adding chitosan NPs on the mechanical characteristics of acrylic resins has not been investigated yet. Cold-cure acrylic resins are most frequently used in the fabrication of removable orthodontic appliances. Therefore, the present study aimed to investigate the effects of chitosan NPs on the mechanical properties of a cold-cure orthodontic acrylic resin [21].

## 2. Materials and Methods

In this in vitro study, various weight percentages of chitosan NPs (0.5%, 1%, 2%, and 4%) were added to the polymer powder of a commercial cold-cure acrylic resin (Acropars, Marlic Co., Tehran, Iran). Acrylic resin, without chitosan NPs, was also used as the control. The polymer powder and monomer liquid were manipulated, according to the manufacturer’s instructions. The flexural strength, compressive strength, Vickers microhardness, and impact strength tests were performed for all five groups:

Group 1: acrylic resin (control group)
Group 2: acrylic resin with 0.5% chitosan NPs
Group 3: acrylic resin with 1% chitosan NPs
Group 4: acrylic resin with 2% chitosan NPs
Group 5: acrylic resin with 4% chitosan NPs.

### 2.1. Preparation of Chitosan NPs

Low-molecular-weight chitosan (Sigma-Aldrich, St. Louis, MO, USA) was dissolved in an acetic acid (1.0%) solution. Next, a sodium tripolyphosphate solution was added to the chitosan solution and stirred, with pH adjusted to nine using sodium hydroxide (NaOH) (Merck, Darmstadt, Germany). Finally, the precipitate was lyophilized to obtain chitosan NPs.

### 2.2. Flexural Strength

**Figure 1:**
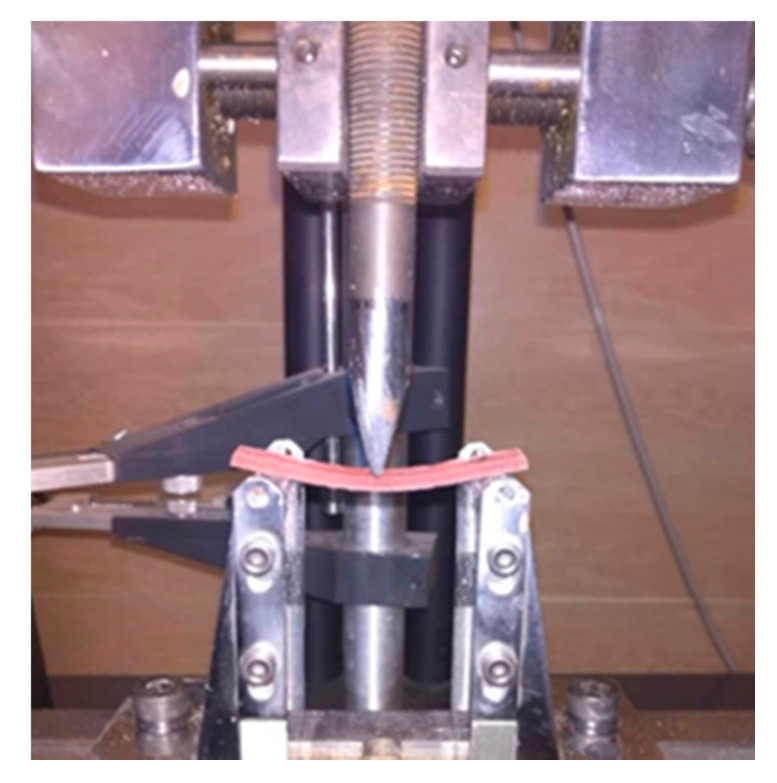
Flexural strength test.

Ten bar-shaped specimens (65 mm × 10 mm × 3 mm) were fabricated per group, using a stainless steel mold. The polymer powder and monomer liquid were manipulated and then the mixture is placed in the mold. When setting of acrylic specimens is completed, desired dimensions are obtained. The samples were stored in distilled water at 37 °C for 48 h. The flexural strength of the specimens was measured according to ISO 20795-1, using a universal testing machine (STM20, SANTAM, Tehran, Iran) at a crosshead speed of 5 mm/min and a span length of 50 mm (Figure 1). The force causing the specimen fracture was recorded, and the flexural strength was calculated using the following formula:

$$\mathrm{FS}=\frac{3\mathrm{Fl}}{2{\mathrm{wh}}^{2}}$$

where F is the load at fracture, l is the distance between the supporting points, w is the specimen width, and h is the specimen height.

### 2.3. Compressive Strength

**Figure 2:**
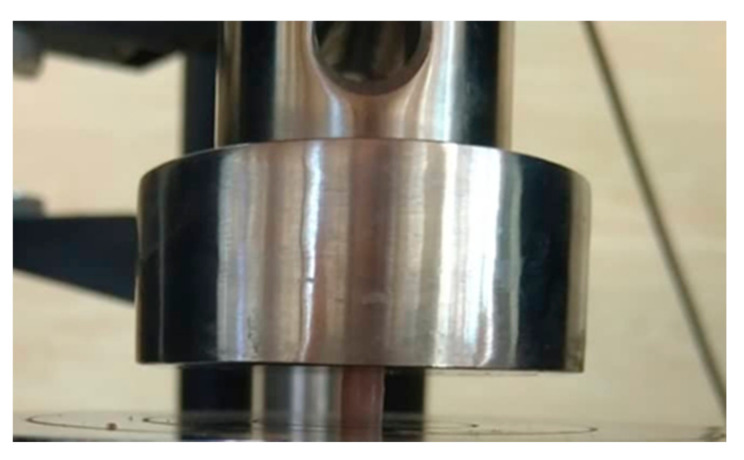
Experimental set up for compressive strength test.

According to the ASTM D695-02a (ISO 604) standard, 23 compressive strength test samples were prepared in this study. A mold (height of 6 mm and diameter of 4 mm) was used to prepare the cylindrical samples for each group. The samples were stored in deionized water for 48 h and then subjected to a compressive strength test in a universal testing machine (STM20, SANTAM, Tehran, Iran) at a crosshead speed of 5 mm/min (Figure 2). The compressive strength was calculated using the following equation:

$$\mathrm{CS}=\frac{P}{\pi r^{2}}$$

where P is the compressive load, and r is the radius of the specimen.

### 2.4. Microhardness

**Figure 3:**
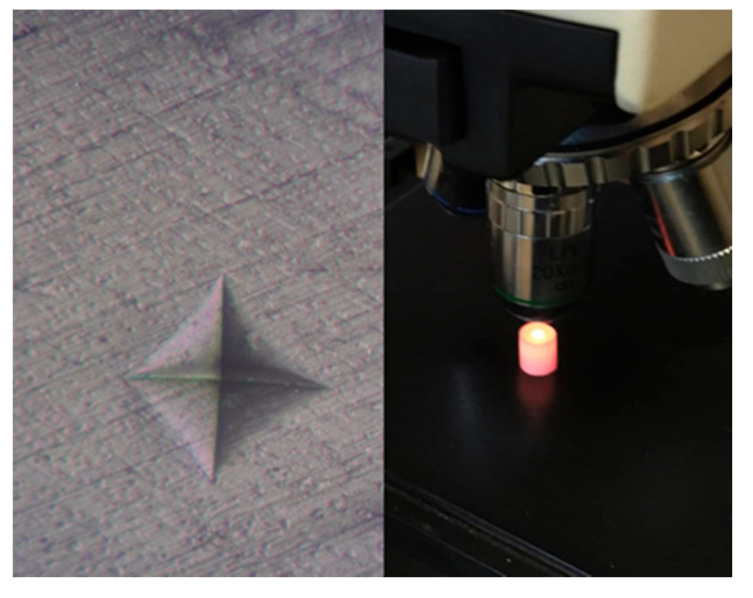
Vickers microhardness test.

A total of 100 disk-shaped specimens (20 per group) were prepared in this study. The specimens were stored in deionized water for 48 h in an incubator at 37 °C. Next, microhardness measurements were carried out, using a Vickers microhardness measurement device (Figure 3). Three indentations were made on each sample, and the mean value was recorded as the Vickers hardness.

### 2.5. Impact Strength

**Figure 4:**
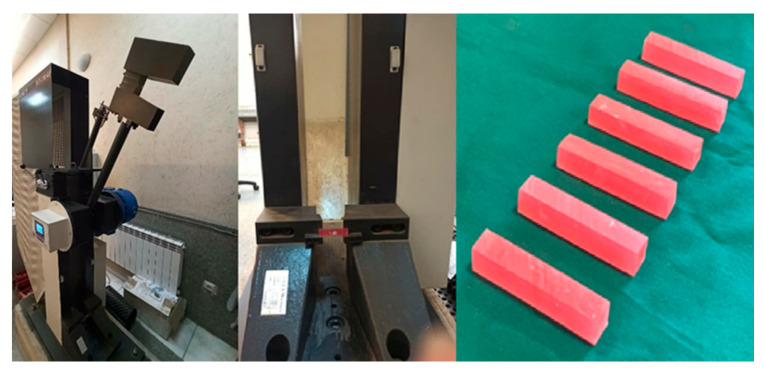
Pendulum Charpy impact testing machine and prepared specimens.

In this study, Charpy impact specimens (10 mm × 10 mm × 55 mm) were prepared. The impact strength was measured, according to the ASTM D-256 standard [22], using a pendulum Charpy impact testing machine (SIT-20E-SANTAM, Tehran, Iran). The specimens were stored in distilled water at 37 °C in an incubator for 48 h. Then, each sample was placed horizontally in the machine (a 4-cm distance between the two fixed supports). The pendulum was fallen with a circular motion to strike the opposite side of the notch (Figure 4). The total absorbed energy was determined as the impact strength and digitally recorded.

The statistical analysis was conducted using the SPSS software version 22 (SPSS Inc., Chicago, IL, USA). All data were analyzed using one-way ANOVA and Tukey’s test at a significance level of 0.05.

## 3. Results

### 3.1. Flexural Strength

The results of ANOVA test revealed significant differences in the flexural strength between the groups (Table 1). The results of Tukey’s test also indicated significant differences between the groups (*p* < 0.001). As shown in Table 2, there was no significant difference between group 1 (control), group 2, and group 3, although the flexural strength decreased significantly in group 4 and group 5. In other words, with increasing the concentration up to 1% chitosan NPs, the flexural strength did not change significantly, but in 2% and 4% chitosan NPs, flexural strength decreased compared to other three groups.

### 3.2. Compressive Strength

Regarding compressive strength, significant differences were found between the groups, based on the ANOVA test (Table 3). The results of Tukey’s test also indicated significant differences between the groups (Table 4). Despite the lower compressive strength in group 2 and group 3 as compared to the control group (group1), the differences between group 2 and the control group (*p*-value = 0.935) and between group 3 and the control group (*p*-value = 0.368) were not statistically significant. However, in group 4 and group 5, the compressive strength significantly decreased.

### 3.3. Microhardness

The microhardness of each group is shown in Table 5. According to the results of ANOVA test, the microhardness was not significantly different between the five groups (*p* > 0.05), and addition of chitosan NPs exerted no significant negative effects on the microhardness.

### 3.4. Impact Strength

Table 6 presents the impact strength (mean and standard deviation) of all groups. The ANOVA test showed no significant differences in terms of the impact strength between the groups (*p* > 0.05), and modification of acrylic resin with chitosan NPs up to 4% had no significant negative effects on the impact strength.

## 4. Discussion

In this study, various mechanical properties of a cold-cure orthodontic acrylic resin, containing chitosan NPs, were evaluated, including the flexural strength, compressive strength, microhardness, and impact strength. There are very few studies investigating the mechanical properties of modified acrylic resins. Flexural strength is one of the most important properties of acrylic resins, which has been investigated in most studies on acrylic resin modification. The present results showed that adding up to 1% (*w/w*) chitosan to acrylic resin had no significant negative effects on its flexural strength, while 2% and 4% (*w/w*) chitosan NPs decreased this parameter.

Chitosan in acrylic resin may act as an impurity in the poly(methyl methacrylate) matrix, which usually decreases the flexural strength in acrylic resins [13]. On the one hand, chitosan may have adverse effects on the degree of conversion in polymerization and lead to an increase in the amount of residual monomer that acts as a plasticizer [23]. On the other hand, chitosan NPs may agglomerate, and the agglomerated particles can act as stress concentration centers in the acrylic resin matrix [24]; overall, these factors might decrease the flexural strength. However, no studies have yet examined the effects of chitosan NPs on the flexural strength of acrylic resin. Moslehifard et al. [25] observed that incorporation of 1 wt% TiO_2_ NPs had no significant effects on flexural strength, compressive strength, and impact strengths of the heat-cure acrylic resin. Their concentrations (0.5%, 1%, and 2%) were similar to our study. Sodagar et al. [14] investigated the effects of TiO_2_ and silicon dioxide (SiO_2_) NPs on the flexural strength of cure acrylic resins. Their results showed that incorporation of TiO_2_ and SiO_2_ NPs at concentrations of 0.5% and 1% (*w/w*) exerted adverse effects on the flexural strength.

Moreover, Ellakawa et al. [26] found that the flexural strength increased significantly after incorporation of aluminum oxide (Al_2_O_3_) into a heat-cure acrylic resin. Alhareb et al. [27] also reported that addition of alumina/zirconia (Al_2_O_3_/ZrO_2_) to a heat-cure acrylic resin improved its flexural strength. Moreover, Al-Harbi et al. [28] demonstrated that addition of 0.5% (*w/w*) nanodiamond to a heat-cure acrylic resin significantly increased its flexural strength; however, the flexural strength decreased by increasing the concentration of chitosan NPs (1% and 1.5%).

In the present study, similar to the trend of flexural strength changes, the compressive strength of acrylic resin significantly decreased with 2% and 4% (*w/w*) chitosan NPs. Hamedi-Rad et al. [29] found that incorporation of 5% (*w/w*) AgNPs increased the compressive strength of heat-cure acrylic resins. In another study, Ghaffari et al. [30] reported that acrylic resin with 0.2% and 2% AgNPs had a significantly higher compressive strength as compared to unmodified acrylic resins. Moreover, Abdulridha et al. [31] found that the compressive strength of both cold-cure and heat-cure acrylic resins increased after the incorporation of TiO_2_ NPs.

Hardness is an important indicator for predicting the wear of dental materials, including acrylic resins. A low surface hardness influences the surface roughness and causes an increase in the plaque retention, pigmentation, and eventually weakness of mechanical properties, compromising its longevity and aesthetic appearance [32]. The results of the current study showed that addition of chitosan NPs had no significant adverse effects on the microhardness of acrylic resin. In this regard, Vojdani et al. [33] investigated the effects of adding 0.5–5% (*w/w*) Al_2_O_3_ on the hardness of a heat-cure acrylic resin. They found that hardness significantly increased after incorporating 2.5% and 5% (*w/w*) Al_2_O_3_.

Generally, removable orthodontic appliances may be fractured when suddenly struck or accidentally dropped. Therefore, acrylic resins should have an adequate impact strength to increase their durability and longevity. Our results revealed that modification of acrylic resin with chitosan NPs up to 4% had no significant negative effects on the impact strength of acrylic resin. Al-Harbi et al. [28] observed that addition of nanodiamond decreased the impact strength of heat-cure acrylic resins. Moreover, the results of a study by Ghahremani et al. [34] showed that color-modified acrylic resins, reinforced with 1 wt% TiO_2_, had a significantly higher impact strength as compared to the conventional acrylic resin. However, few studies have evaluated the impact strength of modified acrylic resin, and most of these studies have only investigated the flexural strength.

One limitation in this study was that the study was performed in vitro, and thus, did not represent complete oral conditions. In oral cavity, the mechanical forces and stresses are different from the in-vitro situation where specimens are exposed to each condition separately. Moreover, specimen configuration was standardized for each mechanical tests and did not demonstrate the actual geometry of a removable orthodontic appliance. For more meaningful results, future studies must be performed more closely mirror the in vivo situation.

## 5. Conclusions

Considering the limitations of this in vitro study, it can be concluded that addition of chitosan NPs up to 1% (*w/w*) had no significant negative effects on cold-cure acrylic resin’s mechanical properties, including flexural strength, compressive strength, microhardness, and impact strength. However, further research is required to investigate other important aspects of modified acrylic resins, such as antimicrobial activity, colorimetric properties, and cytotoxicity.

## Figures and Tables

**Table 1:** Mean and standard deviation of the flexural strength in five different groups (*p* < 0.05).

Groups	*n*	Mean (MPa)	Standard Deviation (MPa)
Acrylic resin(control)	10	64.1	12.41
Acrylic resin + 0.5% chitosan NPs	10	60.4	7.04
Acrylic resin + 1% chitosan NPs	10	52.6	12.29
Acrylic resin + 2% chitosan NPs	10	46.9	11.25
Acrylic resin + 4% chitosan NPs	10	39.3	9.14

**Table 2:** Post hoc Tukey’s multiple comparison test between groups for flexural strength.

(I) Group	(J) Group	*p*-Value
Group 1 (Control)	Group 2	0.939
	Group 3	0.140
	Group 4	0.008
	Group 5	0.000
Group 2	Group 3	0.497
	Group 4	0.056
	Group 5	0.001
Group 3	Group 4	0.763
	Group 5	0.051
Group 4	Group 5	0.472

**Table 3:** Mean and standard deviation of the compressive strength in five different groups (*p* < 0.05).

Groups	*n*	Mean (MPa)	Standard Deviation (MPa)
Acrylic resin(control)	23	92.61	22.86
Acrylic resin + 0.5% chitosan NPs	23	88.78	14.79
Acrylic resin + 1% chitosan NPs	23	83.74	12.48
Acrylic resin + 2% chitosan NPs	23	79.04	14.93
Acrylic resin + 4% chitosan NPs	23	71.35	15.71

**Table 4:** Post hoc Tukey’s multiple comparison test between groups for compressive strength.

(I) Group	(J) Group	*p*-Value
Group 1 (Control)	Group 2	0.935
	Group 3	0.368
	Group 4	0.040
	Group 5	0.000
Group 2	Group 3	0.839
	Group 4	0.274
	Group 5	0.005
Group 3	Group 4	0.871
	Group 5	0.089
Group 4	Group 5	0.515

**Table 5:** Mean and standard deviation of the Microhardness in five different groups (*p* > 0.05).

Groups	*n*	Mean (VHN)	Standard Deviation (VHN)
Acrylic resin(control)	20	17.50	2.19
Acrylic resin + 0.5% chitosan NPs	20	17.37	1.17
Acrylic resin + 1% chitosan NPs	20	16.93	1.18
Acrylic resin + 2% chitosan NPs	20	16.76	1.30
Acrylic resin+ 4% chitosan NPs	20	15.91	2.67

VHN: Vickers Hardness Numbers.

**Table 6:** Mean and standard deviation of the impact strength in five different groups (*p* > 0.05).

Groups	*n*	Mean (J/m^2^)	Standard Deviation (J/m^2^)
Acrylic resin(control)	6	2875	487
Acrylic resin + 0.5% chitosan NPs	6	2769	749
Acrylic resin + 1% chitosan NPs	6	2644	596
Acrylic resin + 2% chitosan NPs	6	2531	380
Acrylic resin + 4% chitosan NPs	6	2389	122

## Data Availability

Data is contained within the article.
